# Ammonium sulfate provides an efficient method for isolating small extracellular vesicles from human biofluids

**DOI:** 10.1038/s41598-026-55129-0

**Published:** 2026-05-31

**Authors:** Qimeng Li, Zinan Guo, Yu Li, Menglu Zhang, Yunxia Yao, Xuemin Wang, Lifang Zhao, Yanning Cai

**Affiliations:** 1 Department of Central Laboratory, Xuanwu Hospital of Capital Medical University, Beijing, 100053 China; 2 Department of Neurobiology, Xuanwu Hospital of Capital Medical University, Beijing, 100053 China; 3 Neuromedical Technology Innovation Center of Hebei Province, The First Hospital of Hebei Medical University, Shijiazhuang, 050000 Hebei China

**Keywords:** Small extracellular vesicles isolation, Plasma, Astrocyte derived extracellular vesicles, Ammonium sulfate, Biological techniques, Biotechnology, Nanoscience and technology

## Abstract

Small extracellular vesicles (EVs) are nanosized vesicles (< 200 nm) secreted from various tissues, including the central nervous system (CNS), into diverse biofluids. Due to their ability to carry molecular cargo that reflects the physiological state of their parental cells, small EVs represent promising diagnostic carriers for neurodegenerative diseases, such as Alzheimer’s disease (AD) and Parkinson’s disease (PD). While biofluid-derived small EVs offer a “liquid biopsy” solution, their clinical translation is severely hindered by the limitations of conventional isolation methods, which are often time-consuming, costly, or yield low purity. Building upon the “ExoPRISM” (Exosome Precipitation by Ionic Strength Modulation) framework, we developed and validated an optimized, cost-effective ammonium sulfate (AS)-based pipeline for small EVs isolation. We have refined and established the optimal AS ratio specifically for plasma and further demonstrated its feasibility for isolating small EVs from saliva. Subsequently, we introduced a targeted purification step using glutamate aspartate transporter (GLAST) antibodies to specifically isolate central nervous system (CNS)-derived EVs. This step robustly demonstrated that our AS-based approach preserves small EVs integrity and enables the effective isolation of Astrocyte-Derived Extracellular Vesicles (ADEVs) for downstream applications. Finally, the practical utility of this optimized protocol was validated in a clinical cohort. Our findings highlight the robustness, high efficiency, and significant translational potential of this AS-based method for the early and accurate diagnosis of neurodegenerative diseases. We optimized the AS concentration for small EVs precipitation, ultimately identifying 2.66 M as the optimal working concentration. This method demonstrated remarkable time efficiency, completing the entire isolation process in approximately 90 min. Furthermore, it only requires centrifugation at 12,000 xg, significantly enhancing its practicality in clinical diagnostic scenarios. To expand its potential application scope, we also tested the method’s efficacy on other bodily fluids and found that the concentration of 2.66 M was equally effective in isolating small EVs from saliva. Comparative analyzes against the commercially available ExoQuick kit demonstrated that our AS-based precipitation method achieved comparable efficacy in isolating small EVs from the plasma of AD patients and in purifying ADEVs. Furthermore, comprehensive validation using nanoparticle tracking analysis (NTA), electron microscopy, and detection of canonical small EVs markers (CD63, CD9) confirmed the functional equivalence of the two isolation methods. The cumulative evidence from this study firmly establishes the AS-based small EVs isolation protocol as a viable and robust efficacy comparable to commercially available kits. This method confers three distinct advantages: enhanced temporal efficiency, reduced cost burden, and minimal interference with downstream assays, rendering it highly amenable to clinical implementation. Notably, it enables the effective isolation of small EVs from plasma, a conventional biofluid widely utilized in clinical diagnostics, as well as from saliva. These attributes underscore the method’s potential in advancing the early detection of neurodegenerative disorders. Looking ahead, the versatility of this approach suggests its applicability across a diverse array of biological specimens, thereby facilitating the expansion of small EVs-based diagnostic research and clinical practice.

**Supplementary Information:**

The online version contains supplementary material available at 10.1038/s41598-026-55129-0.

## Introduction

Small extracellular vesicles (EVs) are endogenous, double-membraned nanovesicles (30–150 nm)^1^ ubiquitously present in body fluids such as plasma, urine, and saliva. Once regarded merely as cellular debris, they are now recognized as key mediators of intercellular communication owing to three distinctive properties: (1) a protective lipid bilayer that shields cargo from degradation, (2) the capacity to transport bioactive molecules including proteins and microRNAs (miRNAs), (3) excellent biocompatibility with minimal immunogenicity. These attributes have positioned small EVs as highly promising tools for clinical diagnostics and targeted therapeutic delivery^2^.

In clinical diagnostics, small EVs have shown remarkable potential across diverse pathologies^3,4^. In oncology, plasma exosomal programmed cell death ligand 1 (PD-L1) has emerged as a valuable liquid biopsy biomarker for head and neck squamous cell carcinoma (HNSCC), enabling early detection, tumor staging, and assessment of immune microenvironment activation^5^. In neurodegenerative diseases, brain-derived EVs capable of crossing the blood-brain barrier offer a unique window into central nervous system (CNS) pathology. Detection of neuronal markers such as amyloid-β (Aβ), phosphorylated tau (p-tau), and disease-specific miRNAs within these brain-derived EVs has facilitated early diagnosis and longitudinal monitoring of Alzheimer’s disease and related disorders^6–8^.

Despite this potential, the clinical translation of small EVs is currently impeded by the absence of isolation technologies that are simultaneously rapid, scalable, and standardized. Established methods, such as ultracentrifugation (UC) and density-gradient centrifugation (DGC), are notoriously cumbersome (4–16 h) and equipment-dependent, frequently compromising vesicle integrity and yield^9–11^. Conversely, size-exclusion chromatography (SEC), while offering superior purity, is restricted by poor scalability and high operational costs. Even widely used polymer-based precipitation methods (ExoQuick), despite their relative simplicity, remain suboptimal; they involve a comparatively longer workflow (~ 2 h), impose a significant cost burden, and introduce contaminating polymers that interfere with sensitive downstream assays^12–15^.

Furthermore, affinity-based isolation techniques have become highly established in EV-based diagnostics due to their unparalleled specificity. However, these methods are often constrained by low overall yields, high reagent costs, and potential epitope masking, making them suboptimal for the initial bulk isolation from large-volume clinical samples. Therefore, there is an urgent need for a highly efficient, cost-effective, and scalable first-step enrichment strategy.

Recently, precipitation via ionic strength modulation exemplified by the breakthrough “ExoPRISM” method^16^—has emerged as a compelling comparable to overcome these limitations. By utilizing high-ionic-strength electrolytes, particularly ammonium sulfate (AS), this technique facilitates the sequential precipitation of small EVs. This method enables high recovery rates and maintains vesicle biological integrity under mild conditions, offering a practical alternative to conventional isolation techniques.^16^.

In this study, we validated and optimized a rapid small EVs isolation method for human plasma and other biofluids. Building upon the ExoPRISM framework, this refined protocol achieves efficient isolation in under 90 min with minimal instrumentation requirements. Notably, the reliance on AS precipitation maintains high compatibility with downstream multi-omics workflows. Since standard sample preparation for advanced analytical platforms, such as mass spectrometry, inherently incorporates purification and desalting modules, the presence of residual AS does not necessitate extra operational steps. Furthermore, owing to its low molecular weight, AS exhibits superior removal efficiency compared to the macromolecular polymers used in commercial kits, thus minimizing signal interference and ensuring high data fidelity in sensitive downstream assays^17,18^. We further demonstrate its utility for cell-type-specific isolation by combining AS precipitation with immunoaffinity capture using biotinylated anti-GLAST antibodies to enrich ADEVs. Head-to-head comparison with the commercial gold-standard ExoQuick kit revealed comparable performance in yield, purity, and recovery of subtype-specific EVs, at significantly reduced time and cost.

## Materials and methods

### Study design

This study was designed as a multi-phase methodological development and validation project. First, using plasma as the primary matrix, we established the feasibility of the optimized ExoPRISM-based precipitation approach by conducting a comparative analysis between saturated AS and a widely used commercial kit (ExoQuick) for small EVs isolation. Second, we performed a preliminary optimization assay on plasma samples to determine the specific salt concentration required for an optimal balance between yield and purity, identifying 2.66 M as the ideal working concentration. To verify the versatility of this protocol, we extended its application to saliva samples, successfully detecting the transmembrane tetraspanin (CD63), the cytosolic chaperone protein (Hsp70), and cell-origin-specific markers (CD171, CD11b, GFAP). Third, shifting our focus to the isolation of specific vesicle subpopulations, we determined the optimal antibody concentration required for the immunocapture of astrocyte-derived EVs (ADEVs). Fourth, we compared the yield and characteristics of ADEVs isolated from small EVs pre-enriched by either the optimized AS method or ExoQuick. Finally, this protocol was applied to a clinical cohort to isolate ADEVs from the plasma of normal control (NC), mild cognitive impairment (MCI), and Alzheimer’s disease (AD) subjects, facilitating the measurement of Complement Factor D (CFD) protein levels.

### Collection and preparation of biofluid samples

Human biofluid samples were utilized across distinct experimental phases in this study. For initial protocol optimization and preliminary assays, samples were obtained from healthy volunteers (*n* = 2). For subsequent clinical validation and biomarker quantification, retrospectively collected plasma samples were obtained from a well-characterized cohort at Xuanwu Hospital of Capital Medical University, comprising 6 Normal Control (NC) subjects, 8 Mild Cognitive Impairment (MCI) patients, and 8 Alzheimer’s Disease (AD) patients (age range: 55–85 years). All study protocols were approved by the Ethics Committee of Xuanwu Hospital.

#### Plasma processing

Whole blood (10 mL) was collected using EDTA anticoagulant tubes (BD Vacutainer, Cat# 02-657-32) and centrifuged at 1500 xg for 15 min at room temperature to separate plasma from cellular components. The resulting supernatant was aliquoted into 0.5 mL volumes and stored at -80 °C. For small EVs isolation, a starting volume of 250 µL of pre-cleared plasma was utilized to ensure methodological consistency and direct comparability with our previously established preliminary studies.

#### Saliva processing

Saliva samples were collected from volunteers using Salivettes (Sarstedt, Cat# 51.1534) and recovered by centrifugation at 1000 xg for 2 min at room temperature. The saliva was aliquoted into 0.4 mL volumes and stored at -80 °C. For small EVs isolation, an input volume of 400 µL of saliva was utilized. This specific volume was dictated by the predefined minimum aliquot size standardized within our biobank repository; utilizing the entire single aliquot was essential to prevent secondary freeze-thaw cycles, thereby preserving the structural and biological integrity of the clinical samples.

### Preparation of AS solution for small EVs precipitation

Preparation of AS Solution for small EVs Precipitation: Ammonium sulfate (Sinopharm Group, Cat#10002918) was dissolved in 0.05 M Tris-HCl buffer (pH 8.0) supplemented with 1 mM EDTA. To evaluate the optimal precipitation efficiency, a gradient of ten AS solutions was prepared, ranging from 0.76 M to 4.17 M with stepwise increments of approximately 0.38 M (specifically: 0.76, 1.14, 1.52, 1.90, 2.28, 2.66, 3.04, 3.42, 3.80, and 4.17 M). The solutions were heated to 60 °C for 30 min to ensure complete dissolution, subsequently cooled to room temperature, and sterilized by filtration through a 0.22 μm membrane to obtain the final isolation reagents.

### Isolation of small EVs from plasma

Cryopreserved plasma samples were thawed at 37 °C for 5 min. Each 0.25-mL aliquot was subjected to centrifugation at 12,000 xg for 15 min at 4 °C to remove cellular debris and large EVs. The resulting supernatant was subsequently treated with 2 µL thrombin (System Biosciences, SBI) for 10 min at 37 °C, followed by a second centrifugation at 12,000 xg for 5 min at 4 °C to deplete fibrinogen and other bulk impurities. The clarified supernatant, hereafter referred to as pretreated plasma, was then used for small EVs isolation.

For the AS-based method, pretreated plasma was combined with an equal volume of an optimized AS isolation reagent and incubated on ice for 30 min. The mixture was centrifuged at 12,000 xg for 5 min at 4 °C, the supernatant was decanted, the final small EVs pellets was resuspended in 250 µL sterile phosphate-buffered saline (PBS) to yield the small EVs fraction. As a comparator, small EVs isolation was also performed on pretreated plasma using the commercial ExoQuick™ precipitation kit (System Biosciences, SBI) in accordance with the manufacturer’s instructions.

### Isolation of small EVs from saliva

Small EVs Isolation from Saliva Cryopreserved saliva aliquots (0.4 mL) were thawed at 37 °C for 5 min. To remove cellular debris and impurities, the samples were subjected to centrifugation at 12,000 xg for 15 min at 4 °C. The clarified supernatant was then combined with an equal volume of the optimized AS isolation reagent (2.66 M) and incubated on ice for 30 min. Following incubation, the mixture was centrifuged at 12,000 xg for 5 min at 4 °C to precipitate the small EVs. The supernatant was decanted, and the final small EVs pellets was resuspended in 250 µL sterile phosphate-buffered saline (PBS).

### Isolation of ADEVs from small EVs

Astrocyte derived small extracellular vesicles were isolated from the small EVs fraction via immunoaffinity capture. Samples were incubated with biotinylated mouse anti-human GLAST antibody (1.5 µg; Miltenyi Biotec, 130-118-984) for 2 h at 4 °C. Subsequently, Streptavidin-Agarose Ultralink resin (10 µL; Thermo Fisher Scientific, 53113)—diluted in 40 µL PBS containing 0.5% BSA—was added and incubated for 30 min at 4 °C with gentle agitation. The resin beads were pelleted by centrifugation at 800 xg for 5 min at 4 °C and washed once with 1 mL of cold PBS. Following a final centrifugation at 4000 xg for 10 min at 4 °C to remove residual buffer, the supernatant was discarded. Bound ADEVs were eluted with 85 µL of 0.05 M glycine-HCl (pH 3.0) and immediately neutralized with 15 µL of 1 M Tris-HCl (pH 8.0). The neutralized samples were lysed with 400 µL M-PER buffer on ice for 30 min, aliquoted 100 µL per tube, and stored at − 80 °C or used immediately.

### Western blotting

Equal amounts of protein samples were denatured and separated by 10% SDS-PAGE (Tris-glycine gels). Following protein transfer, the membranes were incubated with primary antibodies diluted 1:1000 in blocking buffer overnight at 4℃. To qualitatively evaluate characterize the isolated vesicles and verify their cellular origins, the primary antibodies were categorized as follows: (1) transmembrane tetraspanin markers: CD63 (Abcam, Cat# ab134045) and CD9 (Santa Cruz Biotechnology, Cat# sc-13118); (2) cytosolic small EVs markers: Alix (CST, Cat# 2171 S) and HSP70 (Abcam, Cat# ab181606); (3) intracellular negative control: Calnexin (HUABIO, Cat# ET1611-86); (4) co-isolated protein contaminant: ALB (Bioss, Cat# bsm-0945 M); (5) cell-origin-specific markers, which included CD171 indicating neuronal origin (Abcam, Cat# ab208155), CD11b indicating microglial origin (Thermo, Cat# PA5-79532), and GFAP indicating astrocyte origin (Thermo, Cat# 13–0300). After three washes with TBST (Tris-Buffered Saline supplemented with 0.5% Tween 20), the membranes were incubated with HRP-conjugated secondary antibodies (Proteintech, Cat# SA00001-1 & Cat# SA00001-2) at a 1:5000 dilution for 1 h at room temperature. Protein bands were visualized using Western ECL Substrate (Millipore, Cat# WBKLS0500).

### Enzyme-linked immunosorbent assay (ELISA)

The levels of CFD in the isolated ADEVs were quantified using a commercial ELISA kit (Multisciences Biotech, Cat# EK1290) following the manufacturer’s instructions and established protocols. Briefly, 100 µL of standards or diluted ADEV samples (pre-treated with lysis buffer to release internal cargo) were added to the microplate pre-coated with a specific capture antibody and incubated for 2 h at room temperature. After washing away unbound substances, a biotin-conjugated detection antibody was added. Following incubation and washing, Streptavidin-HRP was introduced for signal amplification. The reaction was visualized by adding TMB (3,3’,5,5’-Tetramethylbenzidine) substrate solution, and the color development was stopped by adding a sulfuric acid stop solution. The optical density (OD) of each well was measured at 450 nm using a microplate reader. The final concentrations were calculated based on a standard curve generated with recombinant CFD.

### Morphological observation of small EVs

For morphological observation of the isolated small EVs, 5 µL of the small EVs solution was loaded onto a copper mesh grid and incubated for 1 min. The residual liquid was gently removed using filter paper, and this process was repeated. Uranyl acetate solution was then loaded onto the mesh for 1 min for negative staining. Images were captured using a Transmission Electron Microscope (TEM) (FEI Tecnai Spirit system).

### Nanoparticle tracking analysis (NTA)

The number and size distribution of EVs were analyzed using the NanoSight NS300 system (Malvern Panalytical). Samples were diluted 1:100 in degassed deionized water to a final volume of 1 mL for measurement. Camera settings and other acquisition parameters were carefully adjusted to optimize particle visibility without exceeding a 20% saturation threshold. Data acquisition and analysis were performed using NanoSight NTA 3.2 software (build 3.2.16).

### Resistive pulse sensing (RPS) analysis

The size distribution and concentration of isolated plasma small EVs were characterized at the single-particle level using a Nanocoulter particle size analyzer (Ruixin Technology, China) based on the resistive pulse sensing (RPS) principle. All reagents, consumables, and nanopore chips were proprietary products supplied by the manufacturer. Purified small EV samples were diluted 1:20 with the matched buffer to maintain the particle translocation rate within the instrument’s optimal operational range. Upon sample loading, the system recorded the transient electrical resistance changes (electrical pulse signals) generated as individual particles translocated through the nanopore. Finally, raw data were processed using the accompanying analytical software to accurately determine the absolute particle concentration (particles/mL).

### Ethics statement and informed consent

The experimental protocol and sample collection procedures were approved by the Ethics Committee of Xuanwu Hospital, Capital Medical University (Approved No. 2023 [199]). All methods were carried out in accordance with relevant guidelines and regulations, including the Declaration of Helsinki. Informed consent was obtained from all subjects or their legal guardian(s) prior to participation in the study.

### Statistical analysis

Data normality was confirmed using the Shapiro-Wilk test. Pairwise comparisons of CFD levels between groups (NC vs. MCI, MCI vs. AD, and NC vs. AD) were determined using unpaired Student’s t-tests. All statistical calculations were executed using GraphPad Prism version 10.6.1 (GraphPad Software).

## Results

### Ammonium sulfate facilitates efficient isolation of small EVs from plasma

To evaluate the efficacy of the AS-based method for small EVs isolation, varying concentrations of AS ranging from 0.76 M to 4.17 M were applied to pre-treated human plasma samples. The study aimed at identifying the optimal concentration of AS that ensures maximal small EVs precipitation while minimizing potential issues associated with higher salt concentrations, such as hindered solubility and structural disruption of the targeted EVs.

**Fig. 1:**
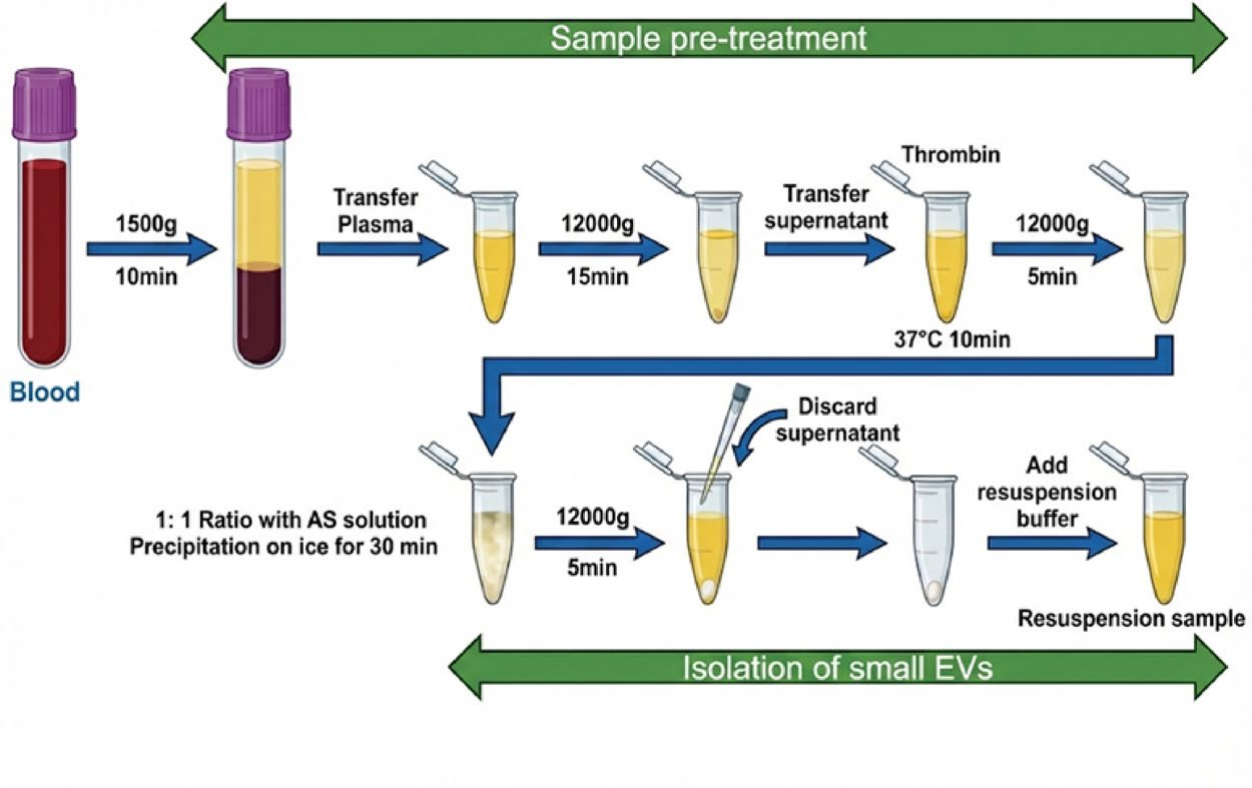
Schematic overview of AS-based method isolation small EVs.

### The AS-based small EVs enrichment protocol exhibits isolation efficiency comparable to that of established commercial kits

We developed a protocol utilizing an ammonium sulfate (AS) solution to isolate small extracellular vesicles (EVs) from biofluids (Fig. 1). To evaluate the isolation efficiency, we quantified the small EV yields and calculated their recovery rates relative to the unpurified plasma baseline (1.35 ± 0.03) x 10^12^ particles/mL across a gradient of AS concentrations, alongside commercial ExoQuick precipitation.

**Fig. 2:**
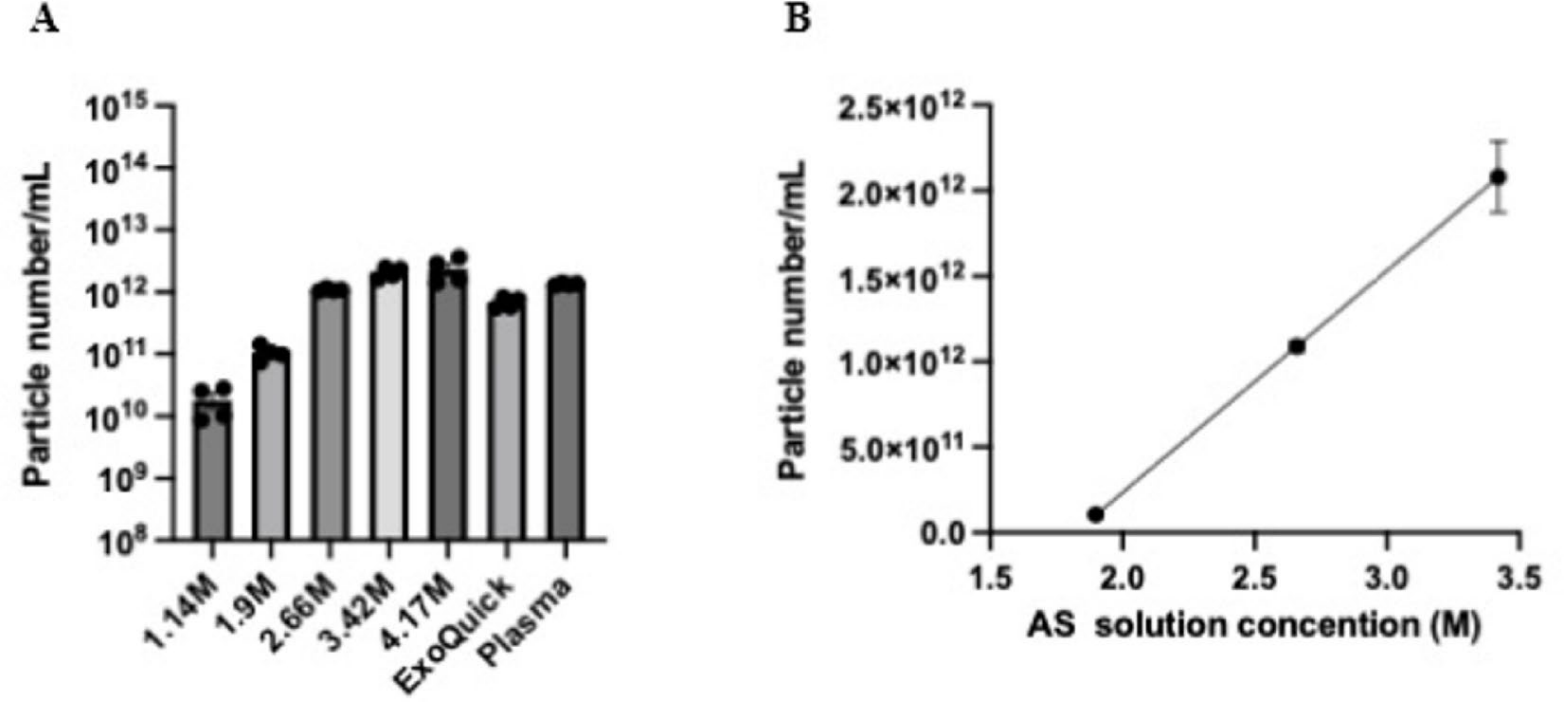
Evaluation of plasma small EV isolation efficiency using different methods and AS concentrations. (**A**) Comparison of small EV isolation efficiency from plasma. The bar chart presents the particle yield (particles/mL) measured directly in the original plasma (baseline), compared to yields obtained using a commercial kit (ExoQuick) and precipitation with varying molarities of AS (1.14 M, 1.9 M, 2.66 M, 3.42 M, and 4.17 M). Error bars indicate the standard error of the mean (SEM), and black dots represent independent biological replicates (*n* = 4). (**B**) Linear regression analysis evaluating the relationship between small EV yield and AS concentration. A strong positive correlation is observed within the 1.9 M to 3.42 M concentration range. The linear fitting equation is Y = 1.3 × 10^12^X − 2.36 × 10^12^, with a coefficient of determination R^2^ = 0.93. Error bars indicate the standard error of the mean (SEM).

As illustrated in Fig. 2A, the recovered particle concentration exhibited a distinct concentration-dependent increase. The yield rose progressively from (1.81 ± 0.5) x 10^10^ particles/mL at 1.14 M (a recovery rate of 1.3%) to a peak of (2.38 ± 0.53) x 10^12^ particles/mL at the saturated 4.17 M concentration (a relative yield of 176.3%). Intermediate AS concentrations produced yields of (1.05 ± 0.14) x 10^11^ particles/mL at 1.9 M (7.8% relative yield), (1.09 ± 0.03) x 10^12^ particles/mL at 2.66 M (80.7% relative yield), and (2.08 ± 0.21) x 10^12^ particles/mL at 3.42 M (154.1% relative yield). In comparison, the standard ExoQuick method yielded (6.81 ± 0.68) x 10^11^ particles/mL, corresponding to 50.4% relative yield.

Notably, the small EV yield deviated from a linear growth trajectory at AS concentrations below 1.9 M and above 3.42 M. However, within the defined 1.9 M to 3.42 M interval, a robust positive linear correlation between the AS concentration and the resulting particle yield was confirmed (R^2^= 0.93), highlighting the predictable nature of this precipitation strategy strictly within this specific concentration window (Fig. 2B).

However, at AS concentrations exceeding 2.85 M, the apparent relative yield of nanoparticles surpassed 100%, reaching a peak of 176.3% at saturation. This paradoxical over-recovery can likely be attributed to the ‘salting-out’ effect occurring at extreme ionic strengths. In a highly complex biofluid such as human plasma, elevated AS concentrations are prone to drive the co-precipitation of abundant plasma proteins and lipoproteins. These non-vesicular components could potentially assemble into nanoscale aggregates that mimic the size distribution of small EVs, which would subsequently lead to an overestimation of the vesicle concentration during analysis.

### Optimization of AS-mediated small EVs isolation from biofluids and antibody-dependent ADEVs purification

**Fig. 3:**
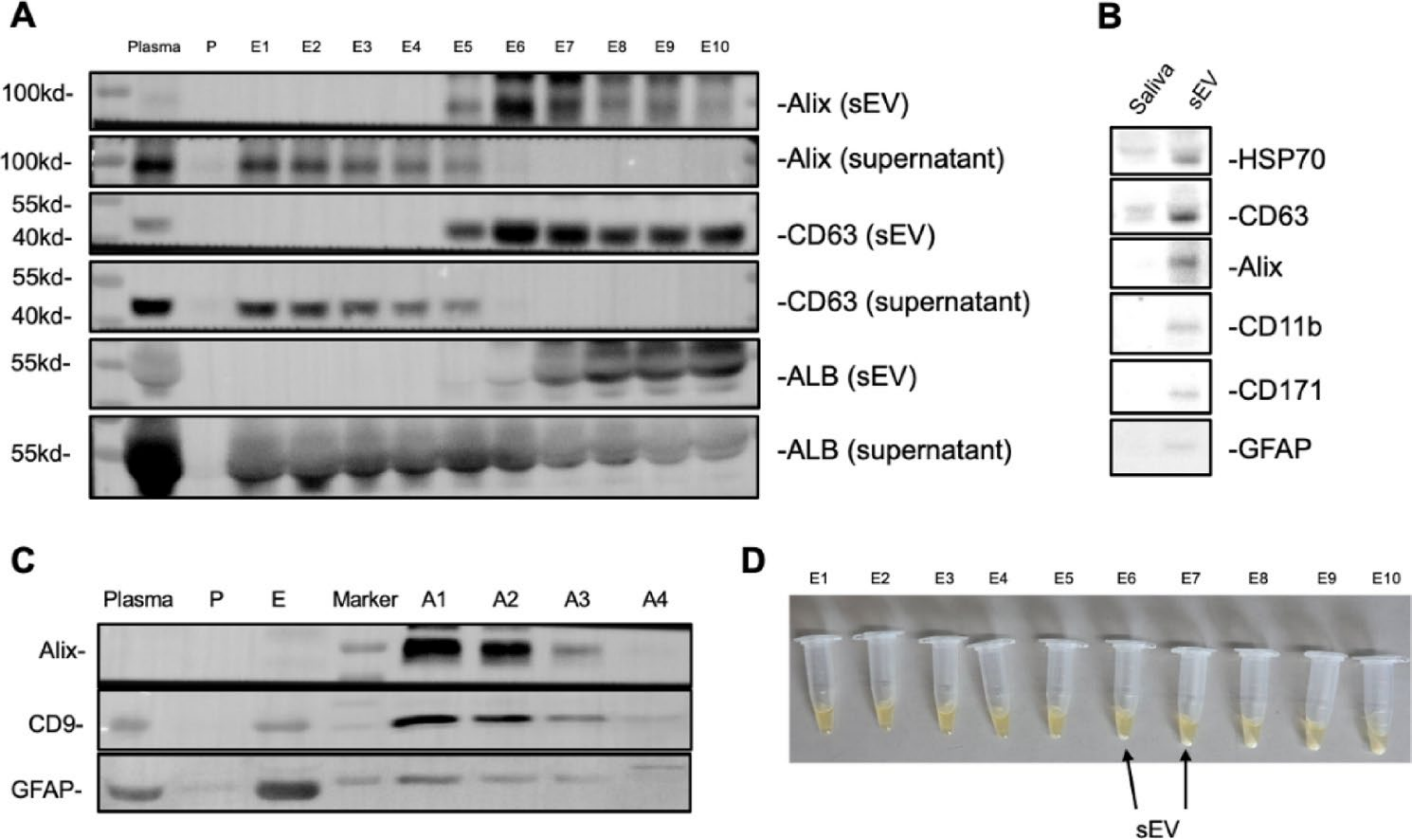
Western blot characterization of small EV isolation and ADEV purification efficiency. (**A**) Western blot analysis of small EV isolation from plasma utilizing a gradient of AS concentrations (E1–E10; ranging from 0.76 M to 4.17 M in increments of ~0.38 M). The large EV pellet (P) serves as a negative control for small EV specificity. Detection of core markers (Alix: 95 kDa, CD63: 30–65 kDa, ALB: 55 kDa) is shown. (**B**) Validation of salivary small EV isolation. Detection of core small EV markers (HSP70: 70 kDa, CD63: 30–65 kDa, Alix: 95 kDa) and origin-specific markers (CD11b: 165 kDa, CD171: 140–250 kDa, GFAP: 40–50 kDa) confirms the successful recovery of small EVs from saliva. (**C**) Optimization of GLAST antibody concentration for ADEV purification. Small EVs were subjected to immunocapture using a titration of GLAST antibody (A1–A4: 1.5 µg, 1.0 µg, 0.5 µg, and 0.15 µg). Plasma, the large EV pellet (P), and purified small EVs (**E**) are included as reference controls. (**D**) Representative images of AS-precipitated plasma small EVs (E1–E10). Visible small EVs (indicated by arrows) correlate with the Western blot results in (**A**), demonstrating the robustness of the AS-based method for small EV recovery.

To establish optimized protocols for small EVs isolation and the subsequent antibody-dependent purification of ADEVs, Western blotting was employed to qualitatively evaluate the performance of the experimental workflows (Fig. 3).

First, we optimized small EVs isolation from plasma by evaluating a gradient of AS concentrations (E1–E10, ranging from 0.76 M to 4.17 M with equal increments of approximately 0.38 M; Fig. 3A). Immunoblotting for canonical small EVs markers (Alix, CD63) revealed a peaked enrichment in small EVs fraction relative to paired supernatants as AS concentration increased. However, signals corresponding to albumin (ALB), an abundant plasma contaminant, also progressively increased in the small EVs fraction at higher AS levels. This finding indicates that excessively high AS concentrations promote the co-precipitation of contaminating proteins. Consequently, considering the critical balance between vesicle recovery and protein purity, 2.66 M AS (Fig. 3A: E6) was identified as the optimal precipitation concentration. At this condition, the protocol achieved a robust small EVs yield of (1.09 ± 0.03) × 10^12^ particles/mL, corresponding to an 80.7% relative yield, while effectively restricting the bulk co-precipitation of ALB contaminants observed at higher molarities. A large EVs pellet (P) served as a negative control and exhibited negligible signals for small EV markers. Further validation of this large EVs fraction using the marker Calnexin is detailed in the supplementary material (Supplementary Figure S1), collectively confirming the specificity of small EVs enrichment under the tested AS gradient conditions.

Next, to validate the versatility of this isolation approach across distinct biological fluids, salivary small EVs preparation were characterized (Fig. 3B). The robust detection of core small EV markers (CD63, Alix) in the isolated fraction confirmed the successful recovery of salivary small EVs. The concomitant assessment of origin-specific small EV markers (CD11b, CD171, GFAP) further verified the retention of cell-type-specific cargo within the isolated vesicles, underscoring the utility of this method for small EVs isolation from non-plasma biological matrices.

Finally, antibody-mediated ADEVs purification from small EVs was optimized by evaluating a gradient of antibody concentrations (A1–A4; Fig. 3C) to determine the optimal condition. Immunoblots monitoring small EV markers (Alix, CD9) and the ADEVs-associated protein GFAP demonstrated that purification efficiency was directly modulated by antibody concentration. Notably, maximal GFAP signal, accompanied by the consistent retention of small EV markers, was achieved using 1.5 µg of GLAST antibody, thereby establishing this as the optimal concentration for the AS-based protocol. Plasma, the large EVs pellet (P), and purified small EVs (E) were included as reference controls to contextualize and validate the specificity of ADEVs enrichment.

### Multi-dimensional validation of the AS-based method as a comparable to ExoQuick for small EVs isolation

**Fig. 4:**
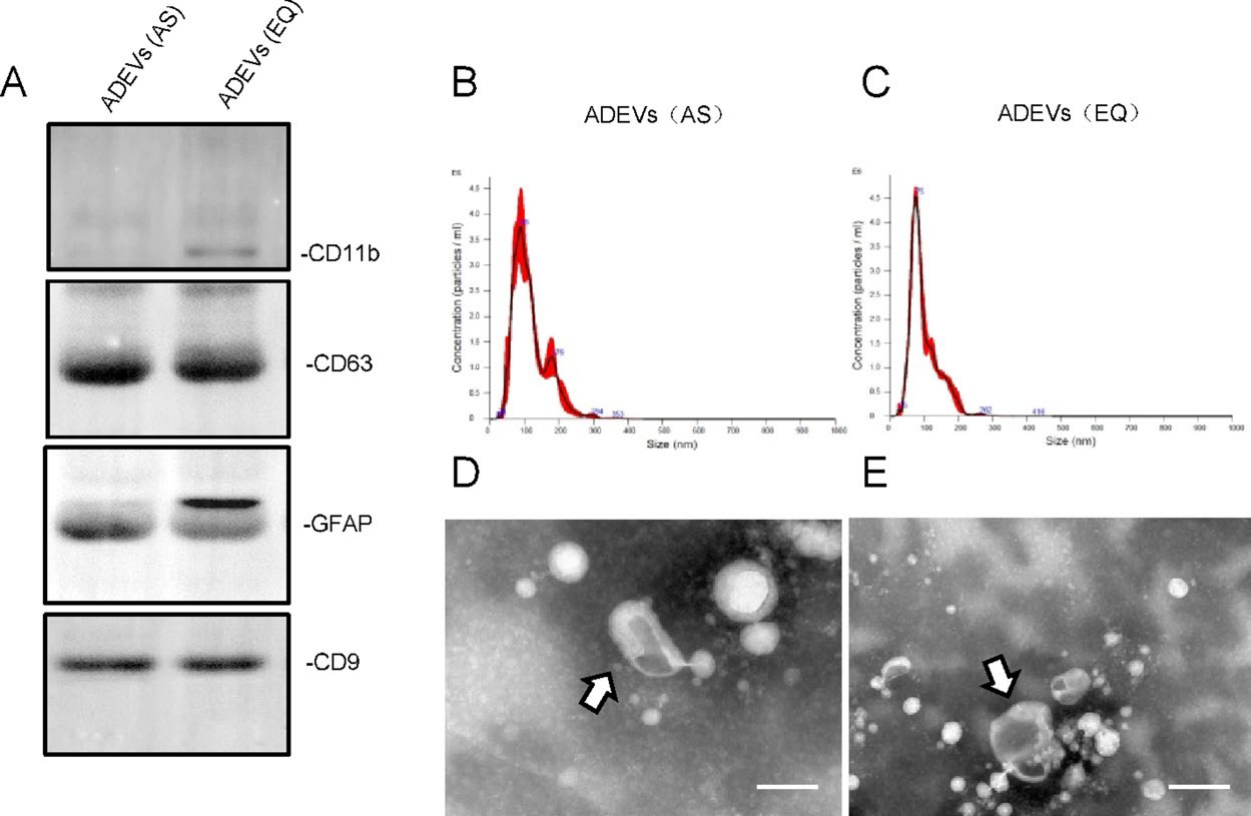
Comparison of ExoQuick and AS-Based Methods for Subsequent Isolation of ADEVs. (**A**) Western blot analysis comparing the expression of small EV markers (CD63: 30–65 kDa, CD9: 25 kDa), the astrocyte-specific marker (GFAP: 40–50 kDa), and the microglial contaminant (CD11b: 165 kDa) in ADEVs isolated using the AS-based method versus the ExoQuick kit. (**B**) Nanoparticle tracking analysis (NTA) illustrating the particle size distribution of ADEVs isolated from plasma using the AS-based method (for the full image, see Supplementary Figure S2). (**C**) NTA illustrating the particle size distribution of ADEVs isolated from plasma using the ExoQuick kit (for the full image, see Supplementary Figure S3). (**D**) Representative transmission electron microscopy (TEM) image displaying the typical morphology of ADEVs isolated via the AS-based method (scale bar: 100 nm). (**E**) Representative TEM image displaying the typical morphology of ADEVs isolated via the ExoQuick kit (scale bar: 100 nm).

Western blot (WB) analysis demonstrated no significant difference in the expression levels of canonical small EVs markers (CD63, CD9) and the astrocyte-specific marker (GFAP) in ADEVs isolated via the AS method (2.66 M) versus the ExoQuick reagent (Fig. 4A). Notably, CD11b—a microglial marker monitored to assess potential contamination—was negligible in both groups. This confirms that when coupled with GLAST antibody-mediated affinity purification (using 1.5 µg antibody per sample), both the AS method and ExoQuick facilitate the highly specific enrichment of ADEVs from small EVs while minimizing microglial-derived impurities (Fig. 4A).

Nanoparticle tracking analysis (NTA) further corroborated the physicochemical equivalence of the AS method and ExoQuick in preserving key ADEVs characteristics. For ADEVs enriched via streptavidin-conjugated Ultralink resin, both isolation protocols yielded vesicles with overlapping particle size distributions. The mean particle diameter ranged from 90 to 120 nm, consistent with the characteristic dimensions of small EVs (Figs. 4B,C).

Transmission electron microscopy (TEM) confirmed that ADEVs isolated via both the AS method and ExoQuick displayed the classic cup-shaped morphology and intact lipid bilayers characteristic of EVs (Figs. 4D-E). No overt structural defects, such as membrane rupture or aggregation, were observed in the AS-isolated group. Furthermore, the paucity of non-vesicular impurities across all samples attests to the high purity achieved by both isolation strategies.

Collectively, these multi-dimensional data establish the AS-based protocol as a comparable to ExoQuick for the isolation of small EVs prior to ADEVs enrichment. Specifically, the AS method preserves small EVs morphology (confirmed by TEM), maintains the integrity of specific biomarkers (validated by WB), and ensures stringent impurity control (evidenced by minimal CD11b levels), thereby satisfying the critical criteria for reliable small EVs-based research applications.

### Expanded sample validation of the AS-based method: efficacy in adevs isolation and potential for clinical application

To rigorously validate the robustness of the AS-based reagent (2.66 M) and isolation protocol, and to assess its applicability for clinical diagnostics, we conducted an expanded sample validation study using human plasma cohorts. In this validation phase, small EVs were isolated from plasma via the AS-based method, followed by the enrichment of ADEVs using a combination of biotinylated GLAST antibody (1.5 µg/sample) and streptavidin-conjugated Ultralink resin (10 µL/sample)—adhering strictly to the optimized protocol established in our pilot experiments.

To evaluate the clinical diagnostic potential of our AS-based isolation method, we quantified CFD levels in the purified ADEVs. CFD was selected as a strategic biomarker because it has been previously validated as a robust indicator for distinguishing healthy individuals from patients with MCI and AD^19^. Our results demonstrate that the AS-isolated ADEVs maintain sufficient integrity for the sensitive detection of such disease-specific markers.

**Fig. 5:**
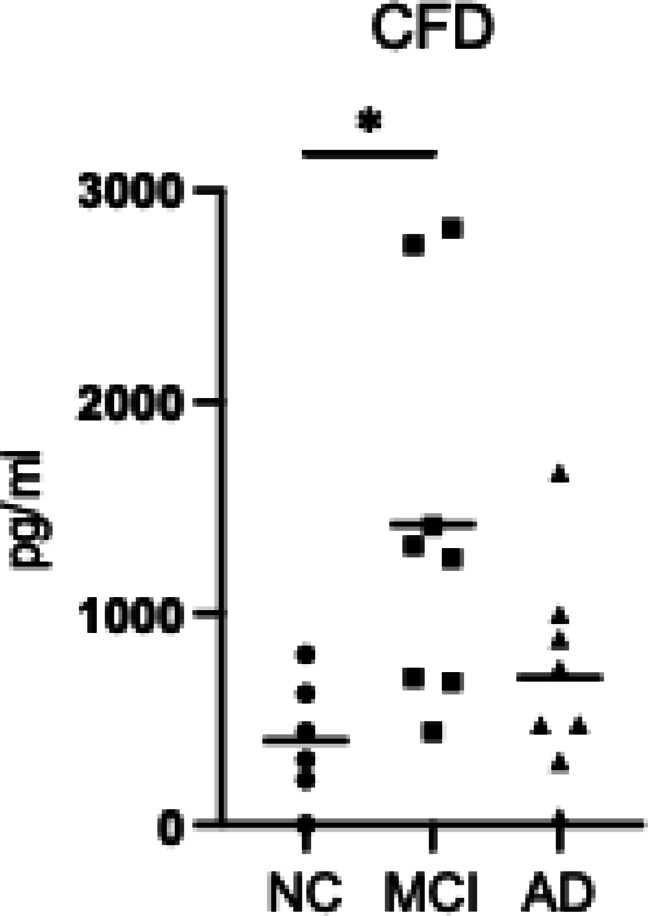
Quantification of complement factor D levels in ADEVs isolated from human plasma. The study cohorts included normal controls (NC, *n* = 6), mild cognitive impairment (MCI, *n* = 8), and Alzheimer’s disease (AD, *n* = 8). Data are presented as mean ± SEM. *P < 0.05.

We subsequently quantified CFD in the isolated ADEVs across three study groups: Normal Control (NC, *n* = 6), Mild Cognitive Impairment (MCI, *n* = 8), and Alzheimer’s Disease (AD, *n* = 8). The measured concentrations were 398.4 ± 118.0 pg/mL (NC group), 1422 ± 321.3 pg/mL (MCI group), and 696.5 ± 177.2 pg/mL (AD group) (data presented as mean ± SEM; Fig. 5).

Our analysis revealed that CFD levels in ADEVs from both MCI and AD patients were elevated compared to the NC group. Notably, the difference between the MCI and NC groups was statistically significant (*P* < 0.05).

## Discussion

Research exploring the use of AS for EV isolation has recently gained significant momentum^20–22^ Notably, a breakthrough study by Sunkara et al. introduced “ExoPRISM”, elegantly demonstrating that modulating ionic strength with electrolytes like AS enables the sequential precipitation of EVs^16^. Their findings established that AS precipitation yields high recovery rates and preserves structural integrity without the need for ultracentrifugation.

In this study, we further refine and advance the AS-based protocol. While previous studies like ExoPRISM validated the precipitation effect of ammonium sulfate on small EVs, we preliminarily established the optimal AS ratio specifically tailored for complex human plasma and further demonstrated its feasibility for isolating EVs from saliva. Consequently, our optimized AS protocol serves as a robust, highly efficient, and potential alternative to commercial polymer-based kits (Table 1). Notably, it exhibits superior temporal efficiency, requiring only approximately 80 min to complete the isolation process, compared to 135 minutes^23^ for the ExoQuick kit. This streamlined workflow represents a significant improvement over both commercial kits and conventional ultracentrifugation methods.

Crucially, our expanded validation using clinical samples (NC, MCI, and AD cohorts) confirmed that the AS method enables the stable isolation of EVs and the sensitive quantification of downstream biomarkers. Specifically, the quantified CFD profiles, characterized by a significant elevation in the MCI group, align closely with previously reported profiles in neurodegenerative pathology^19^. This biological concordance serves as compelling evidence for the reliability of our AS-based isolation method, confirming that it preserves the pathological signatures necessary for accurate clinical assessment. These findings validate the robustness of the method and highlight its significant potential for large-scale clinical applications.

While high-salt precipitation is often associated with non-specific protein co-precipitation, our optimization identified a precise AS concentration (2.66 M) that yields a higher EV recovery while minimizing the precipitation of contaminating plasma proteins (Albumin). This optimized condition ensures that the isolated EVs maintain high purity, as evidenced by the high expression of small EV markers (CD63, CD9) and negligible levels of impurities (Albumin) in our multi-dimensional validation.

Unlike commercial kits that rely on polyethylene glycol (PEG) or other macromolecular polymers^24–26^, our method utilizes AS, a low-molecular-weight inorganic salt. This distinction is critical for downstream applications: residual polymers from commercial kits are difficult to remove and can interfere with sensitive assays such as mass spectrometry. AS can be efficiently removed through routine experimental steps already integral to many workflows, such as the mandatory desalting procedures in mass spectrometry. Consequently, this method minimizes interference with sensitive downstream analyses without introducing additional operational burdens.

The AS-based protocol addresses these bottlenecks by offering a rapid workflow (≤ 90 min) with minimal equipment requirements and negligible reagent costs. This combination of speed, high purity, and economic feasibility significantly enhances the potential for broad clinical accessibility, particularly in resource-limited settings.

Despite these promising results, several practical considerations and limitations of the current study must be objectively acknowledged to guide future applications:

First, certain pre-analytical considerations regarding sample processing warrant discussion. In this study, we utilized retrospectively collected plasma samples from the Xuanwu Hospital biobank. As these samples underwent a standardized single centrifugation step (1500 xg) prior to cryopreservation, they lacked the rigorous two-step centrifugation protocol required to obtain true platelet-free plasma (PFP). Consequently, the potential co-precipitation of platelet-derived extracellular vesicles (EVs) remains a confounding factor inherent to precipitation-based enrichment methods. While the robust expression of canonical small EV markers (CD63, CD9) in our multi-dimensional validation indicates a high yield of genuine vesicles, and our downstream GLAST-based immunoaffinity purification specifically targets astrocyte-derived EVs (ADEVs) to mitigate non-specific interference, we objectively acknowledge that the influence of residual platelet-derived vesicles cannot be entirely excluded. To maximize isolation purity and minimize background interference, we emphasize that future prospective studies must incorporate a mandatory secondary centrifugation step prior to cryopreservation. This pre-freezing step is essential to ensure the complete removal of residual platelets, thereby preventing their activation or lysis during the freeze-thaw cycle, which could otherwise release confounding EVs into the biofluid matrix.

Second, regarding residual salts and downstream multi-omics compatibility: While the reliance on high concentration AS inherently introduces inorganic salts into the final small EVs pellet, this does not necessarily impose an additional procedural burden. Standard pre-analytical protocols for advanced omics platforms, such as protein mass spectrometry^27,28^ and next-generation sequencing (NGS)^29^, intrinsically include a requisite purification step. Because inorganic salts have a substantially lower molecular weight than target macromolecules, AS is far easier to eliminate than the macromolecular polymers used in commercial kits, routinely yielding removal efficiencies of 98%–99%^30^, which drastically contrasts with residual polymers that frequently cause severe signal interference in protein mass spectrometry^31,32^. Interestingly, some literature suggests that, rather than merely acting as a contaminant, moderate amounts of residual AS might even enhance signal sensitivity in certain mass spectrometry applications^33,34^. These potential benefits provide compelling theoretical grounds for utilizing AS as an isolation reagent in omics-centric research. However, to uphold the highest standards of scientific rigor, we recognize that the current study did not directly validate these specific removal efficiencies or evaluate the downstream multi-omics performance of AS-isolated EVs. Such investigations represent a critical next step and will be prioritized in our future studies to provide a more comprehensive evaluation of this method’s utility.

Third, regarding cargo integrity during AS clearance: While traditional desalting procedures carry a potential risk of losing EVs and relevant luminal cargo (miRNAs and proteins), we believe that with the continuous advancements in sample preparation and desalting technologies for mass spectrometry, the removal of small inorganic salts (like AS) is having an increasingly negligible impact on overall sample quality and vesicle integrity^27^. Nevertheless, how this potential cargo loss compares to that of traditional high-purity isolation options (such as SEC or UC) remains to be rigorously quantified in future systematic comparative studies.

Finally, regarding isolation efficiency and inter-batch reproducibility: A critical metric for any novel isolation technique is its consistency across independent isolations. While our current method demonstrates strong potential and comparability to commercial kits in initial research settings, potential differences in isolation efficiency and reproducibility between distinct biological replicates or different operators have not been exhaustively quantified. Claiming absolute suitability for “clinical diagnostics” requires establishing a rigorous coefficient of variation (CV) for EV yield and cargo recovery. The current method has not yet achieved the validated throughput, large-scale inter-batch reproducibility, and extensive performance metrics required for standardized clinical in vitro diagnostics (IVD).

In conclusion, the optimized AS-based precipitation method represents a highly accessible and time-efficient tool for small EVs isolation, particularly advantageous for initial biomarker discovery and resource-limited research environments. Moving forward, to further optimize this strategy and address current purification limitations, we specifically suggest exploring combinatorial approaches. For instance, employing AS precipitation as a rapid, high-throughput primary enrichment step, followed by Size Exclusion Chromatography (SEC) or target-specific immunoaffinity capture, could effectively eliminate co-precipitated proteins and residual salts. Such tandem workflows would seamlessly complement the high-yield nature of AS precipitation with the ultra-high purity of advanced downstream methods. Ultimately, integrating these robust workflows and extensive validations will be crucial to fully unlocking the method’s potential for translational research and future clinical in vitro diagnostics.

**Table 1:** Comparison of AS-based and ExoQuick small EV isolation parameters.

Comparison parameters	AS-based method	Commercial kits (ExoQuick)
Precipitating agent	Low-molecular-weight inorganic salt	Macromolecular polymers
Processing time	~ 80 min	~ 135 min
Reagent cost	Negligible	Expensive
Equipment required	Minimal (Standard benchtop centrifuge)	Minimal (Standard benchtop centrifuge)
Interference removal	Readily eliminated via desalting steps	Notoriously difficult to remove
Yield & Purity	Comparable yield; optimized to minimize albumin contamination	High yield; but prone to polymer and non-specific protein carryover
Main limitations	The impact of residual salt ions on sensitive downstream applications	High-cost limits large-scale use; polymer residue restricts multi-omics

## Conclusion

In conclusion, this study establishes the optimized AS-based protocol as a high-performance, rapid, and cost-effective strategy for small EVs isolation. By identifying the optimal concentration (2.66 M) to ensure minimal impurity co-precipitation, we demonstrate that the AS method offers a promising alternative that overcomes key limitations of existing techniques—specifically regarding processing time, purity, cost, and downstream assay compatibility. The successful extension of this method to the specific enrichment of ADEVs further affirms its utility in specialized diagnostic applications. These findings support the broader adoption of AS-based isolation in both basic research and large-scale clinical validation of small EVs biomarkers.

## Supplementary Information

Below is the link to the electronic supplementary material.

Supplementary Material 1

Supplementary Material 2

Supplementary Material 3

## Data Availability

The datasets generated and/or analyzed during the current study are available from the corresponding author upon reasonable request. Additionally, all relevant experimental data have been deposited in the EV-TRACK knowledgebase (EV-TRACK ID: EV260035) (Van Deun J, et al. EV-TRACK: transparent reporting and centralizing knowledge in extracellular vesicle research. Nature Methods. 2017;14(3):228-32).

## Declarations

### Statement

In the preparation of this manuscript, artificial intelligence models were employed to optimize the linguistic flow of specific passages. Following the utilization of these tsools, a thorough review and refinement process was conducted to maintain scientific accuracy and ensure clarity of expression. The authors assume full accountability for all content presented in the published work.
