# Supplementary material for: Ammonium sulfate provides an efficient method for isolating small extracellular vesicles from human biofluids
Source: Sci Rep. 2026 May 31;16:24866. doi: 10.1038/s41598-026-55129-0 (PMC13457951; doi:10.1038/s41598-026-55129-0)

Origin Blots images
Figure 2

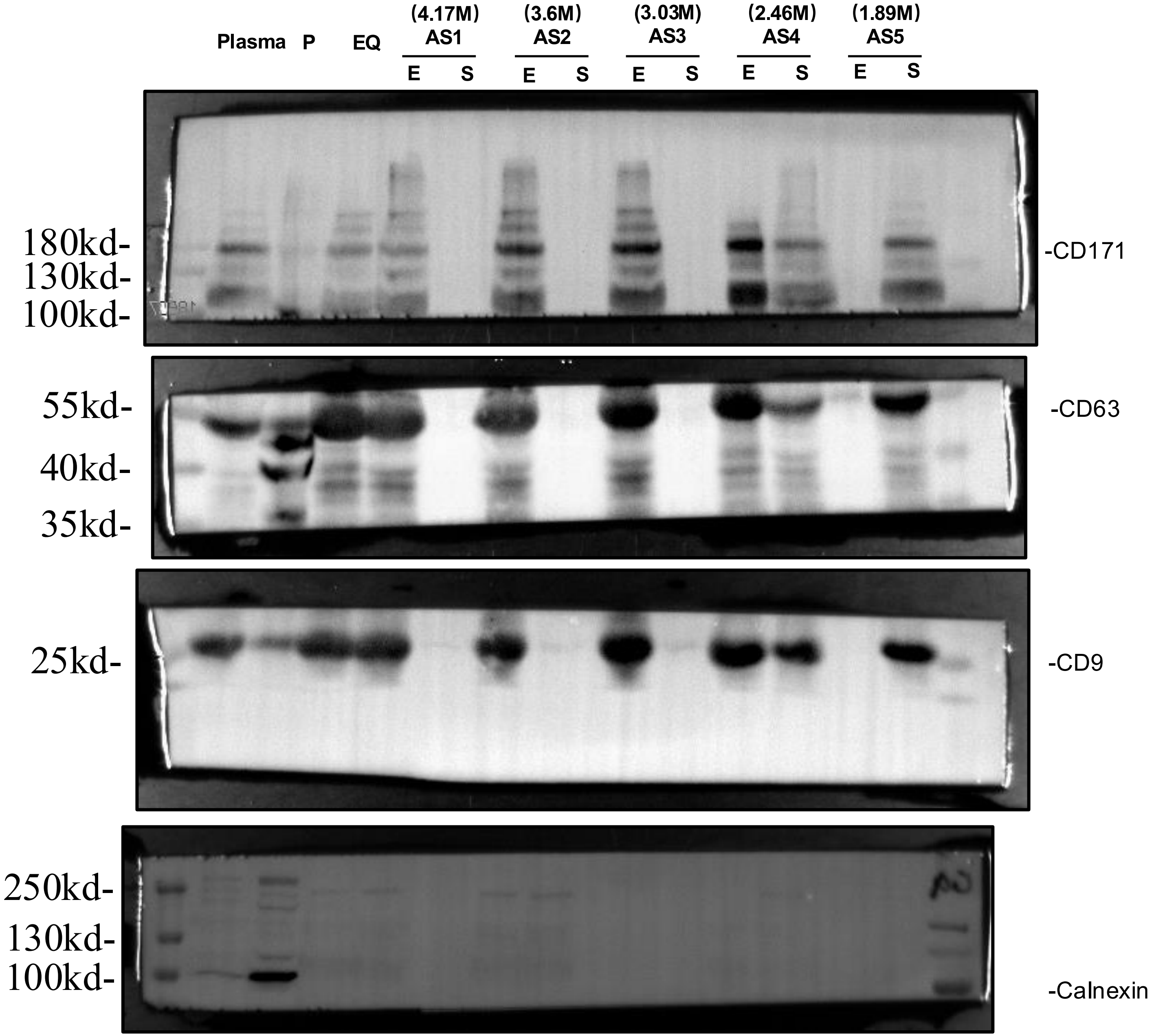

Origin Blots images
Figure 3A

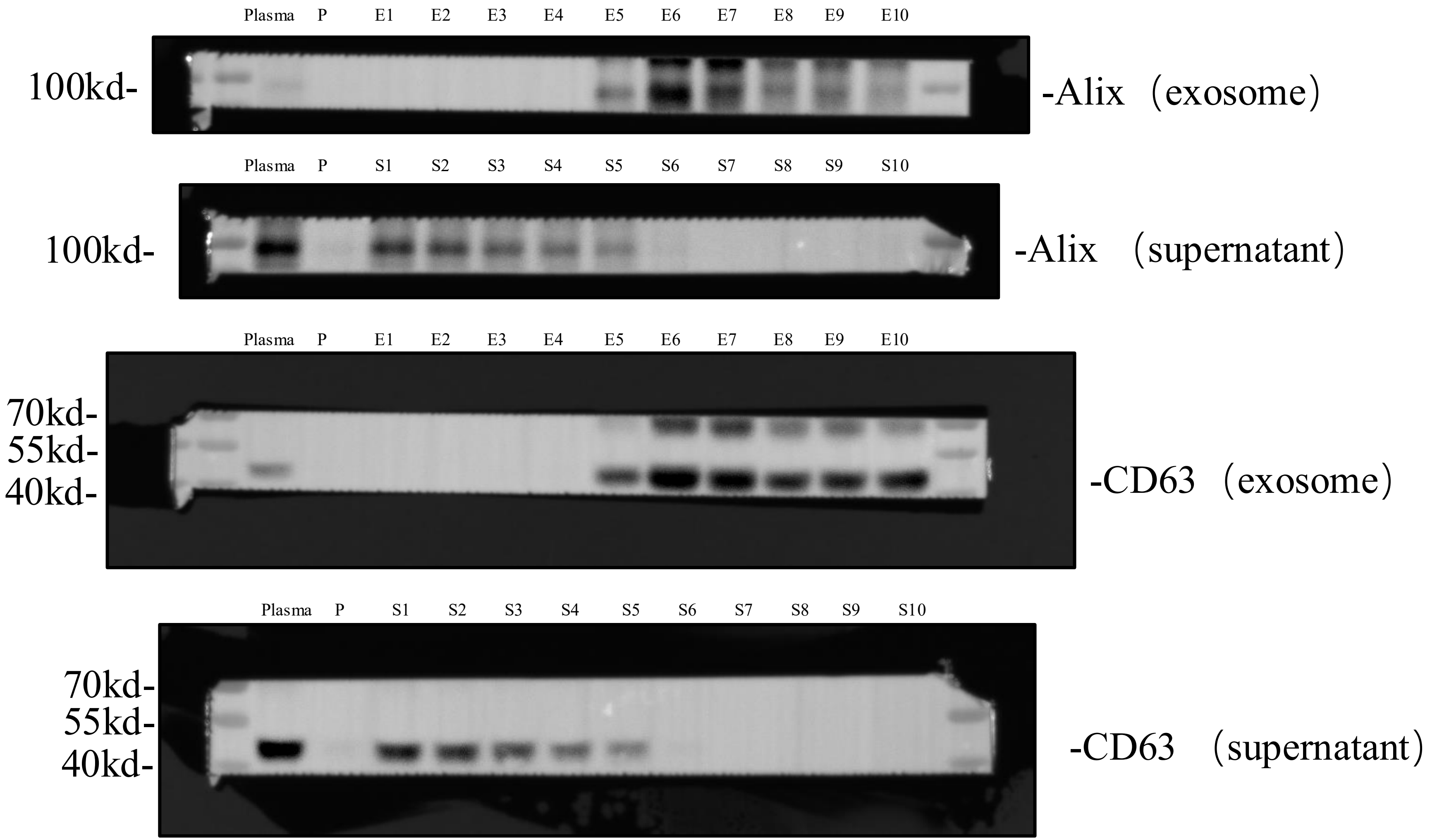

Origin Blots images
Figure 3A

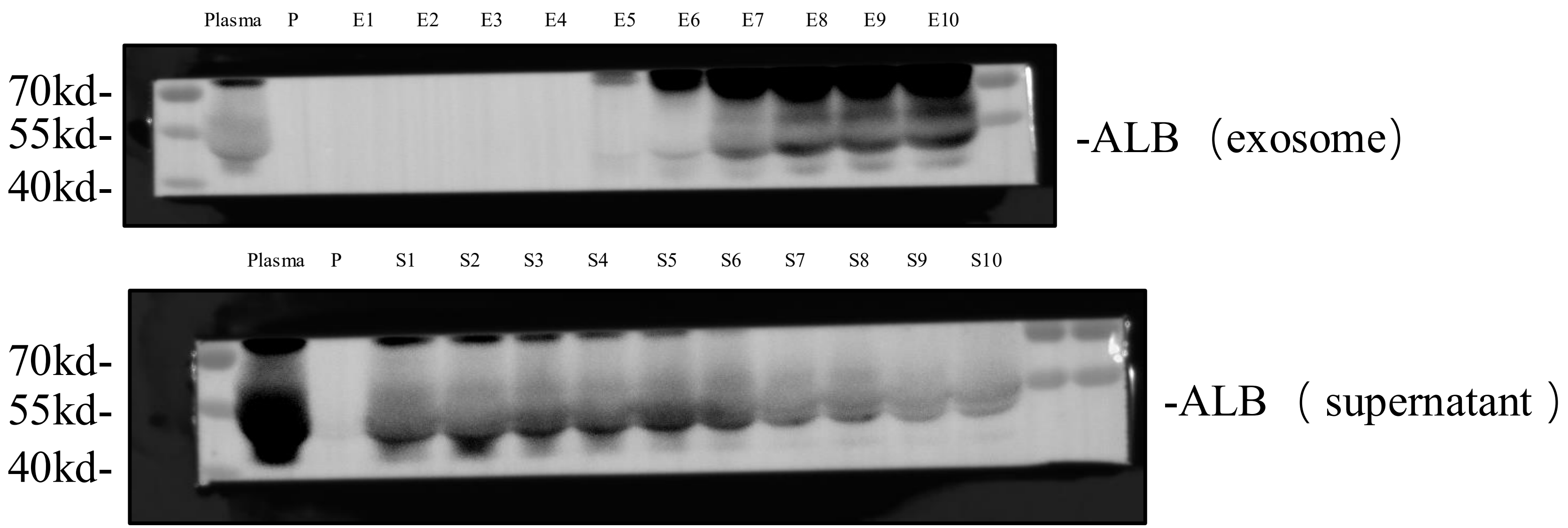

Origin Blots images
Figure 3B

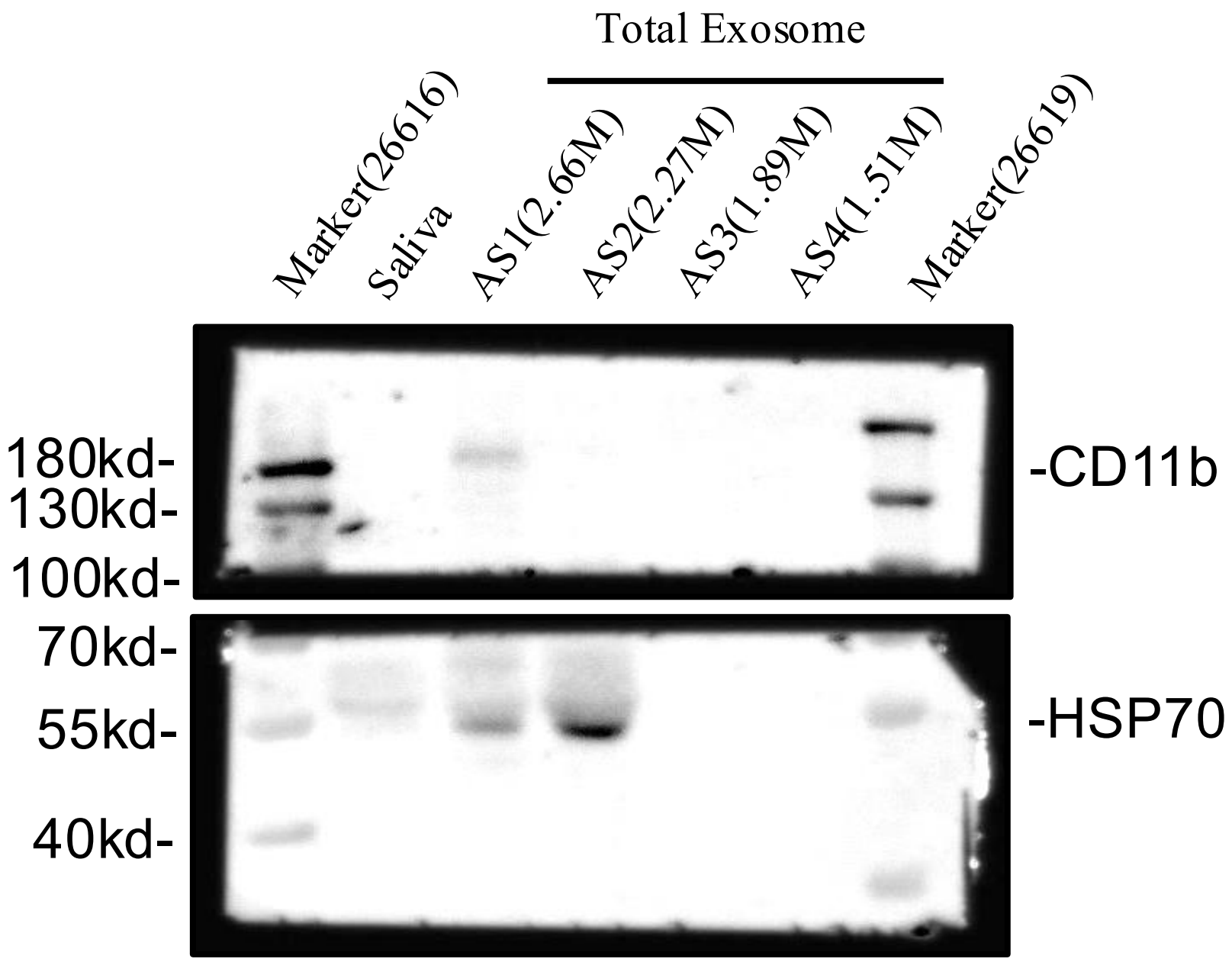

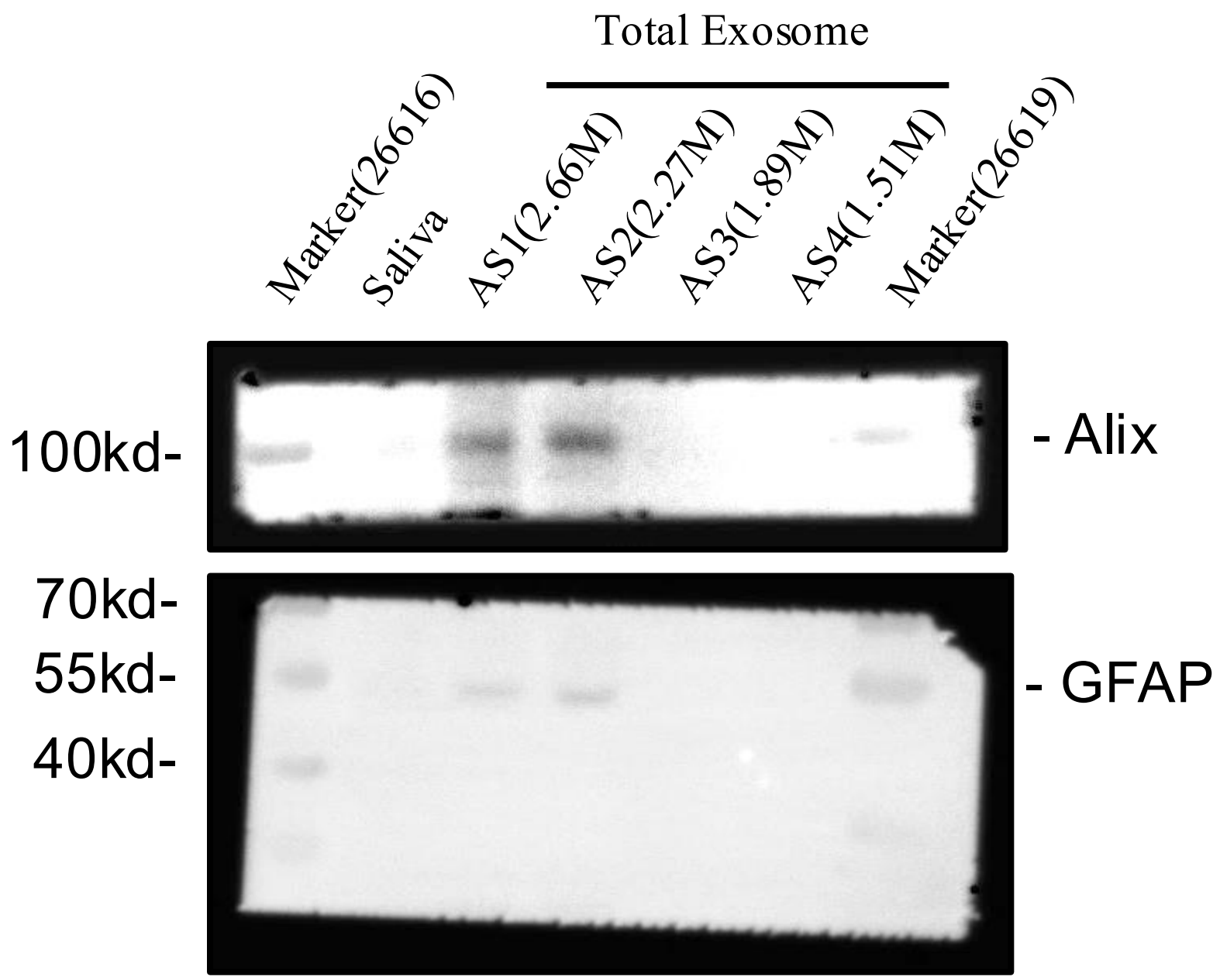

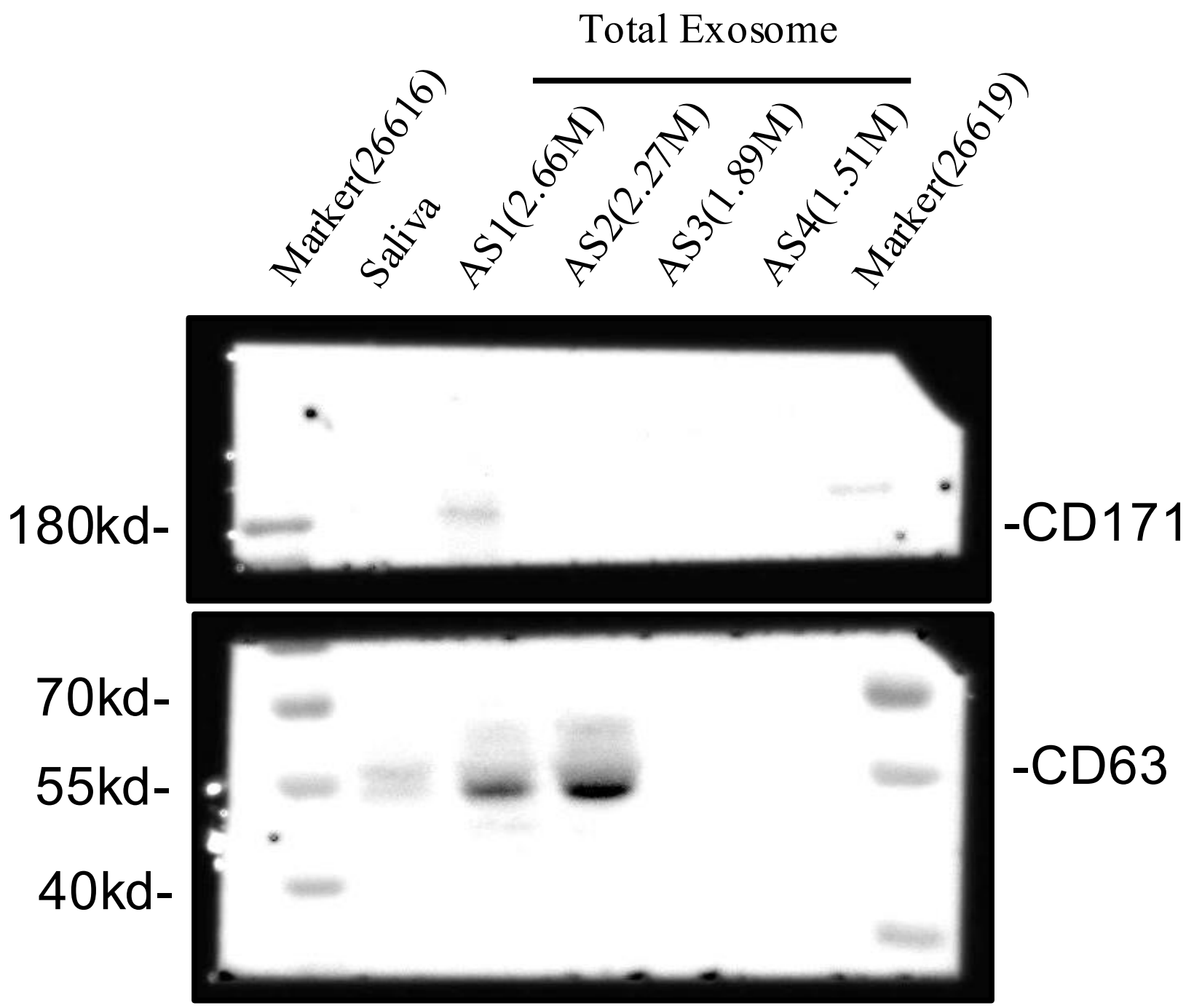

Origin Blots images
Figure 3C

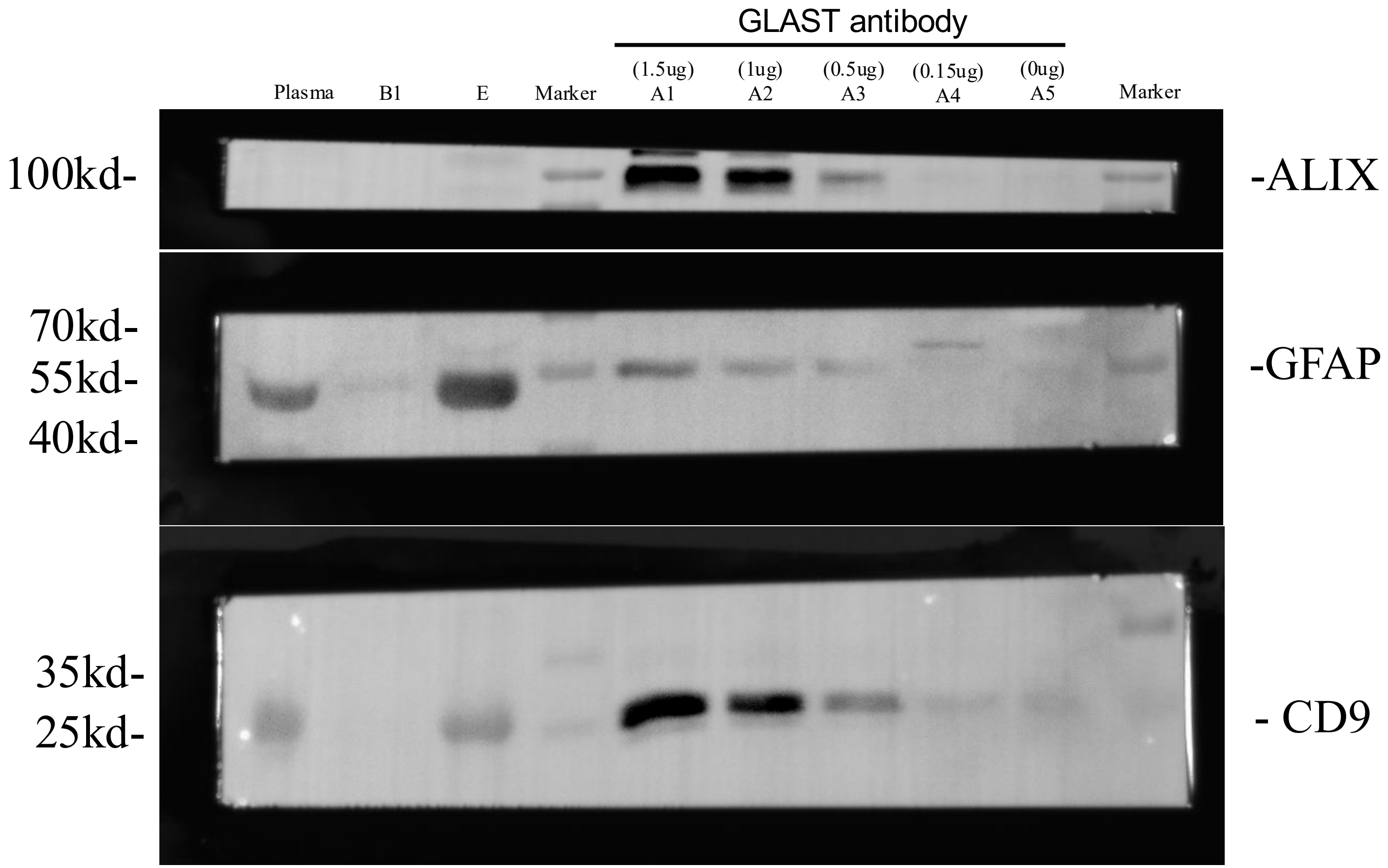

Origin Blots images Figure 4A

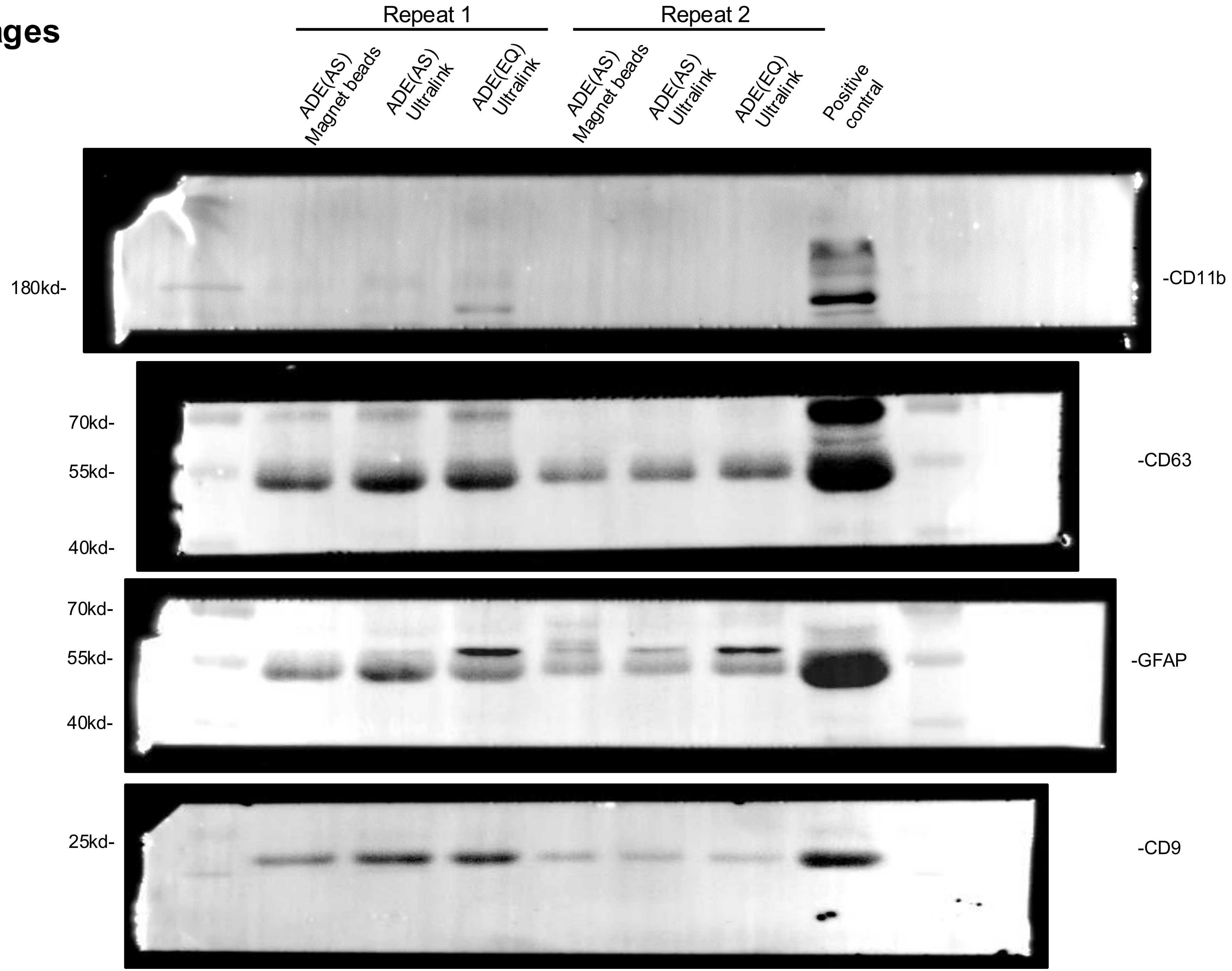

Supplement: Supplementary file 1 [file 41598_2026_55129_MOESM1_ESM.pdf]
