# Supplementary material for: Ammonium sulfate provides an efficient method for isolating small extracellular vesicles from human biofluids
Source: Sci Rep. 2026 May 31;16:24866. doi: 10.1038/s41598-026-55129-0 (PMC13457951; doi:10.1038/s41598-026-55129-0)

Origin Blots images
Figure S1

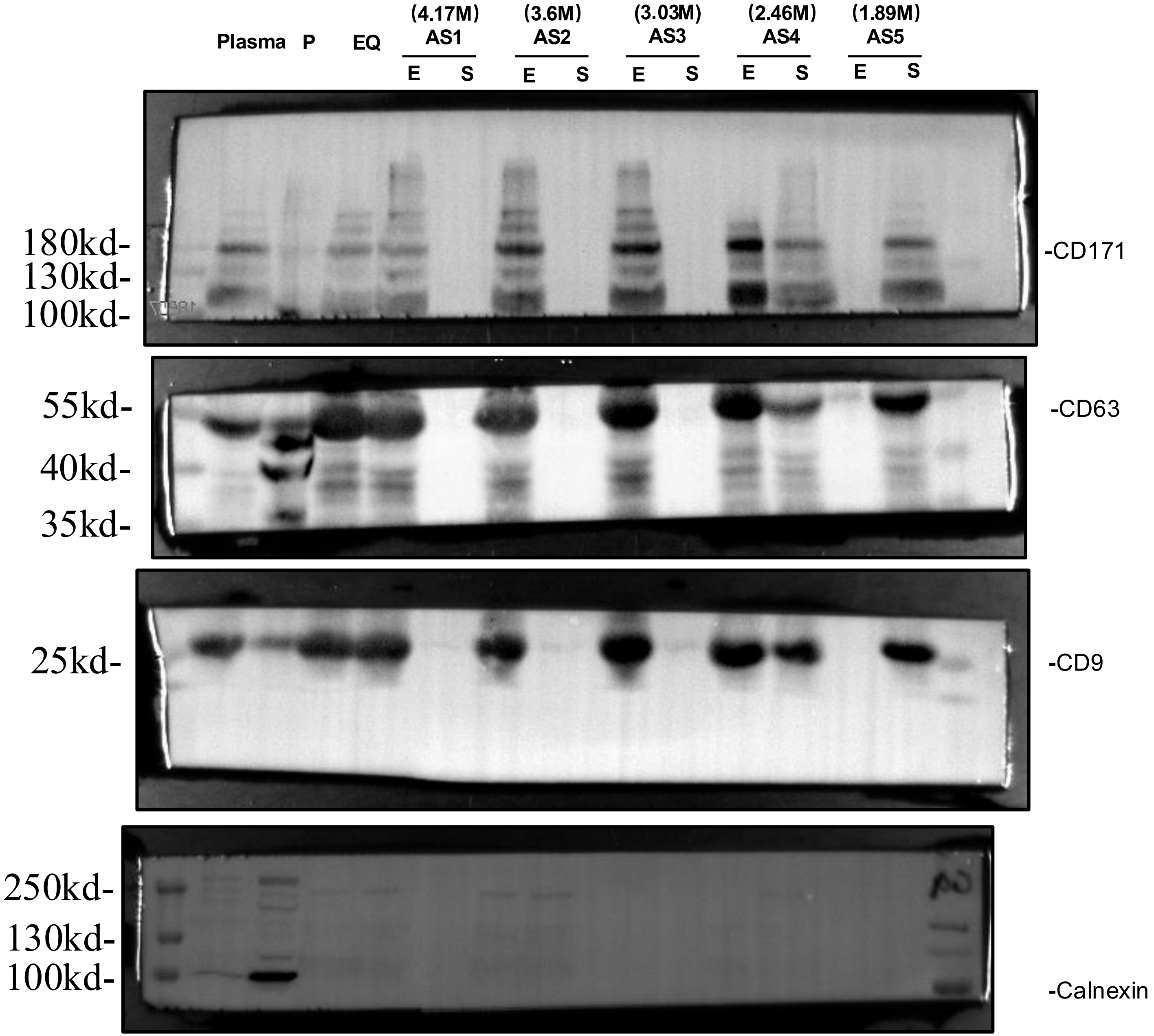

Origin Blots images
Figure 3A

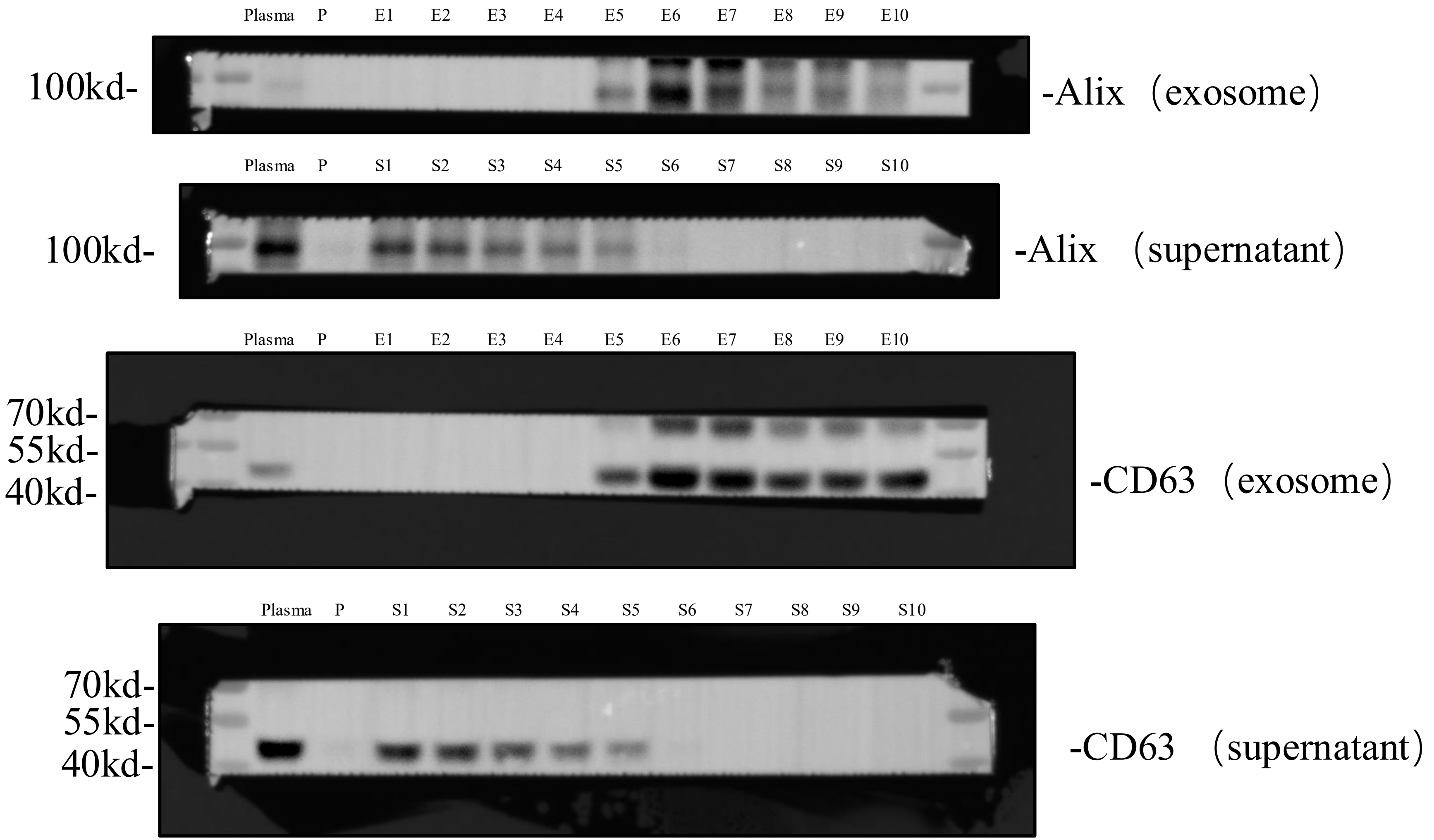

Origin Blots images
Figure 3A

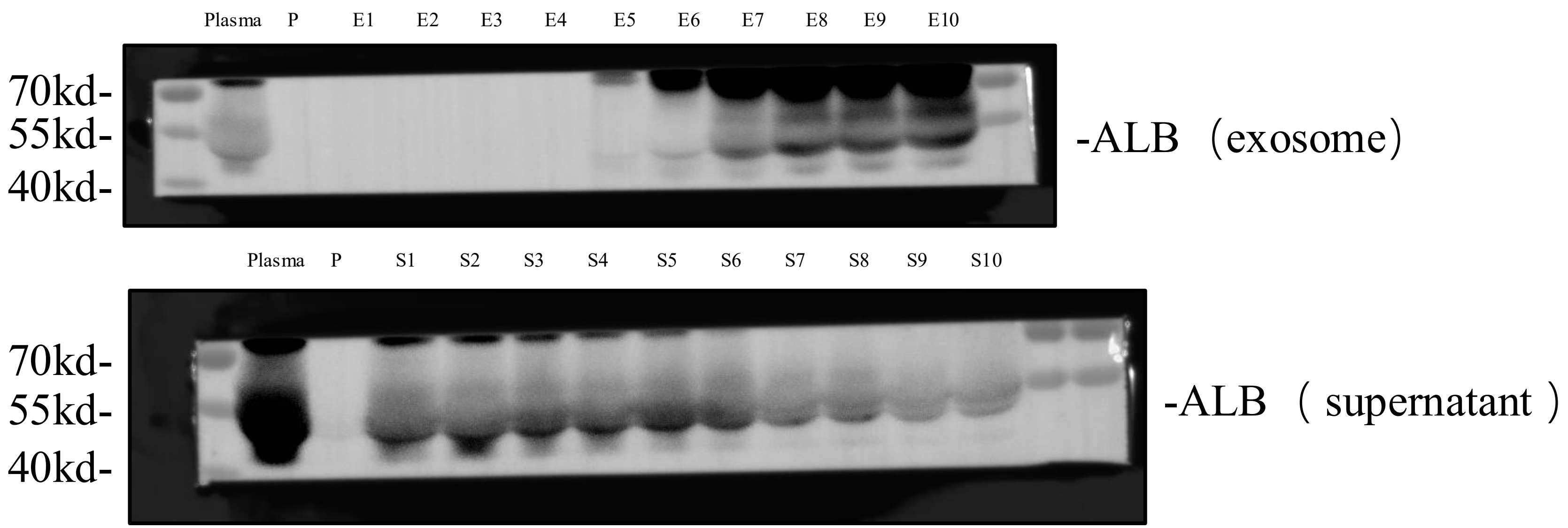

Origin Blots images
Figure 3B

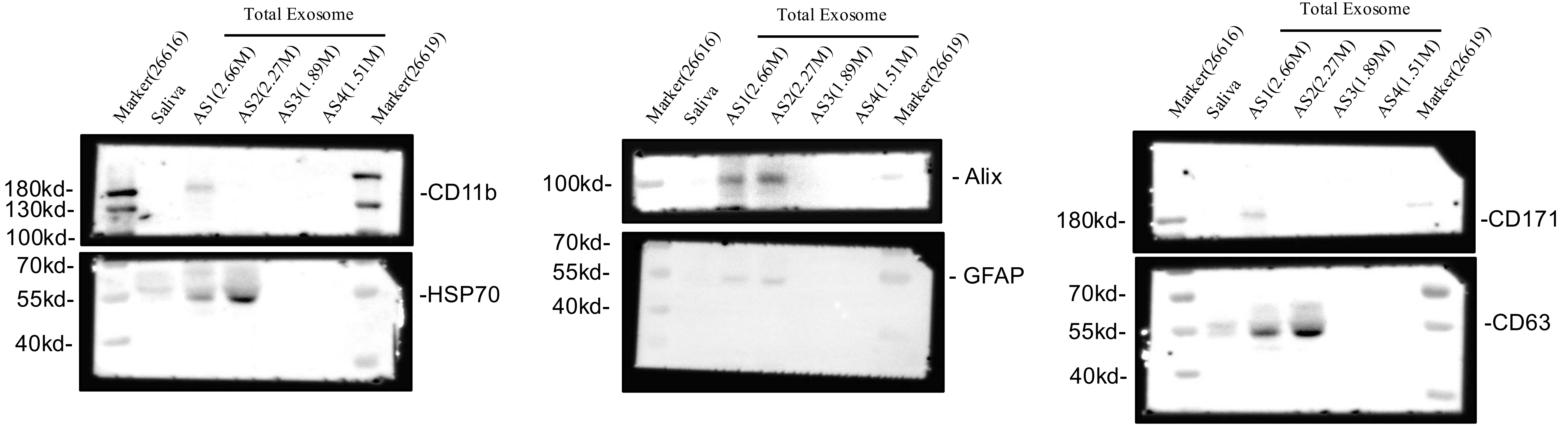

Origin Blots images
Figure 3C

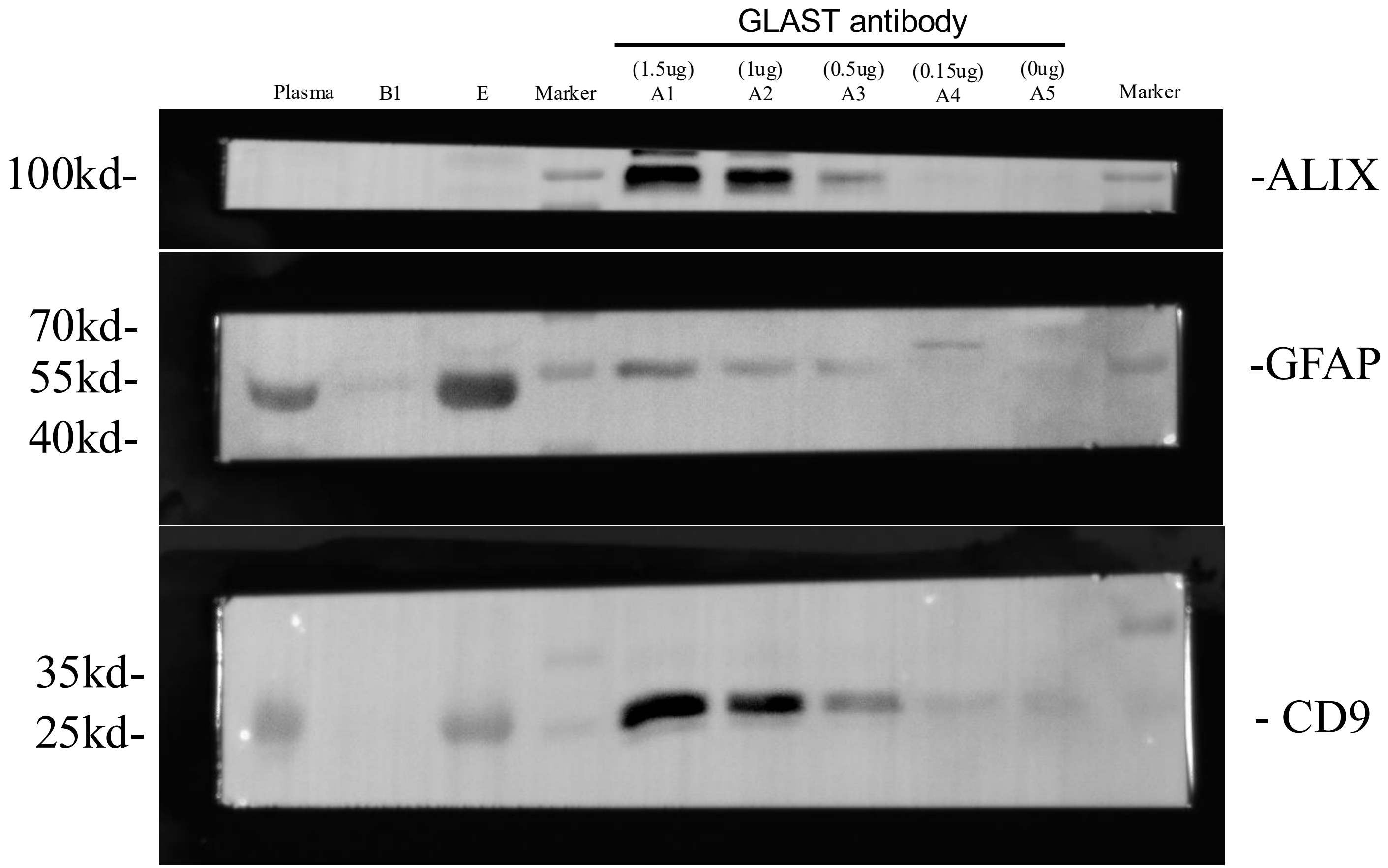

Origin Blots images
Figure 4A

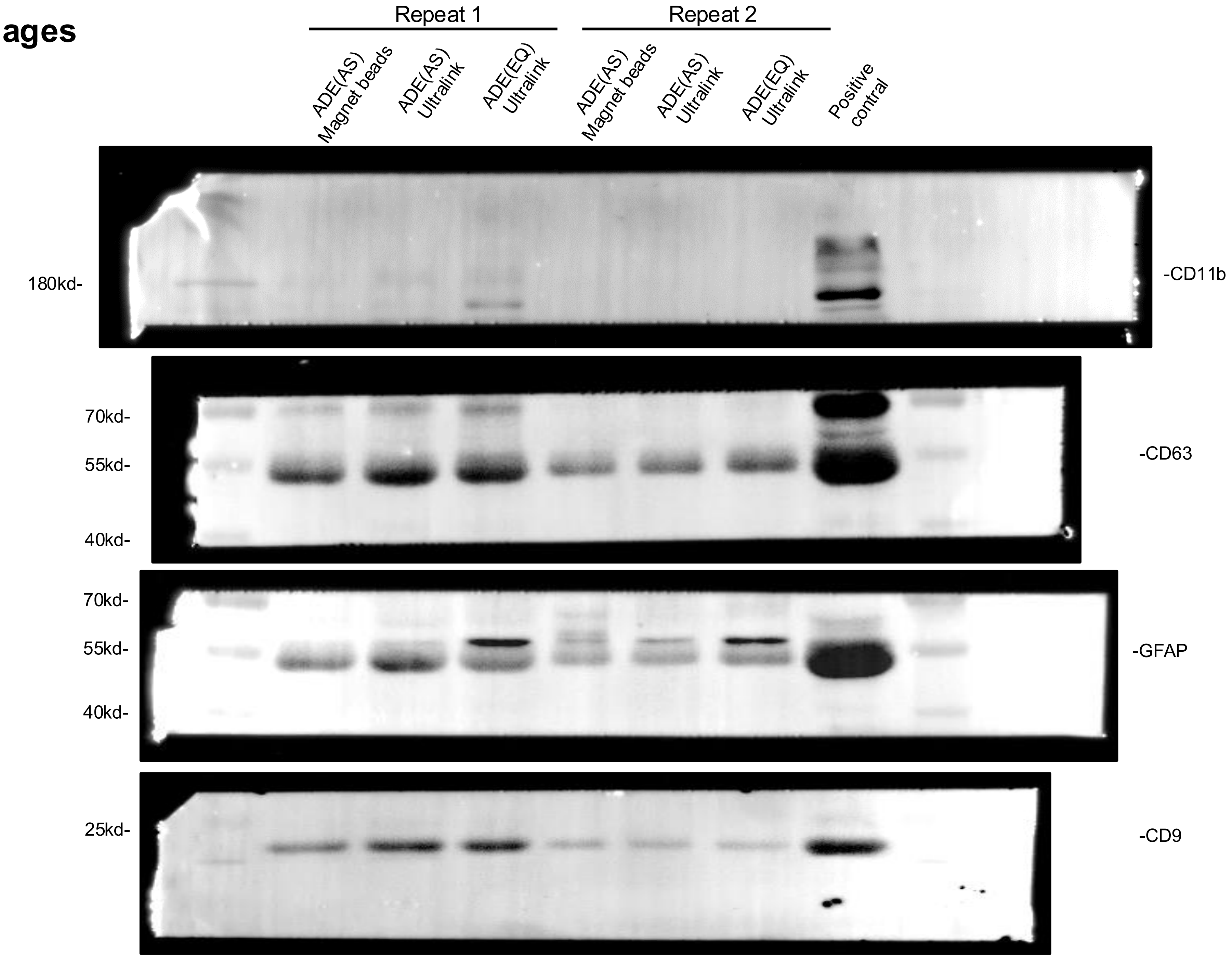

Supplement: Supplementary file 2 [file 41598_2026_55129_MOESM2_ESM.pdf]
