# Supplementary material for: Ammonium sulfate provides an efficient method for isolating small extracellular vesicles from human biofluids
Source: Sci Rep. 2026 May 31;16:24866. doi: 10.1038/s41598-026-55129-0 (PMC13457951; doi:10.1038/s41598-026-55129-0)
Supplement: Supplementary file 3 [file 41598_2026_55129_MOESM3_ESM.docx]

Supplementary

Supplementary Figure S1: Verification of population-specific protein profiles in small and large EV fractions from a single donor.

Figure S1: Western blot analysis was employed to detect the respective biomarkers in both small and large EV fractions, isolated from a single donor using AS-based/ExoQuick protocols (for small EVs) and centrifugal pelleting (for large EVs) (P=Large EVs Pellet, EQ=ExoQuick, AS=Ammonium sulfate solution (4.17 M), E=small Extracellular Vesicle, S=Supernatant).

Supplementary Figure S2-3: Full-scale NTA size distribution profiles of isolated small EVs and ADEVs.

Corresponding full-scale NTA profiles are presented for astrocyte-derived EVs (ADEVs) that were subsequently purified from the initial AS-based method and ExoQuick kit isolated small EV pools via immunoaffinity capture.

Figure S2: Full-size NTA profile for ADEVs isolated from plasma using the AS-based method (corresponding to Figure 4B).

Figure S3: Full-size NTA profile for ADEVs isolated from plasma using the ExoQuick kit (corresponding to Figure 4C).
